# Design Considerations in Development of a Mobile Health Intervention Program: The TEXT ME and TEXTMEDS Experience

**DOI:** 10.2196/mhealth.5996

**Published:** 2016-11-15

**Authors:** Jay Thakkar, Tony Barry, Aravinda Thiagalingam, Julie Redfern, Alistair L McEwan, Anthony Rodgers, Clara K Chow

**Affiliations:** ^1^ The George Institute for Global Health Camperdown Australia; ^2^ Sydney Medical School The University of Sydney Sydney Australia; ^3^ Westmead Hospital Sydney Australia; ^4^ The University of Sydney Sydney Australia

**Keywords:** text message, mobile phone, coronary artery disease, mHealth

## Abstract

**Background:**

Mobile health (mHealth) has huge potential to deliver preventative health services. However, there is paucity of literature on theoretical constructs, technical, practical, and regulatory considerations that enable delivery of such services.

**Objectives:**

The objective of this study was to outline the key considerations in the development of a text message-based mHealth program; thus providing broad recommendations and guidance to future researchers designing similar programs.

**Methods:**

We describe the key considerations in designing the intervention with respect to functionality, technical infrastructure, data management, software components, regulatory requirements, and operationalization. We also illustrate some of the potential issues and decision points utilizing our experience of developing text message (short message service, SMS) management systems to support 2 large randomized controlled trials: TEXT messages to improve MEDication adherence & Secondary prevention (TEXTMEDS) and Tobacco, EXercise and dieT MEssages (TEXT ME).

**Results:**

The steps identified in the development process were: (1) background research and development of the text message bank based on scientific evidence and disease-specific guidelines, (2) pilot testing with target audience and incorporating feedback, (3) software-hardware customization to enable delivery of complex personalized programs using prespecified algorithms, and (4) legal and regulatory considerations. Additional considerations in developing text message management systems include: balancing the use of customized versus preexisting software systems, the level of automation versus need for human inputs, monitoring, ensuring data security, interface flexibility, and the ability for upscaling.

**Conclusions:**

A merging of expertise in clinical and behavioral sciences, health and research data management systems, software engineering, and mobile phone regulatory requirements is essential to develop a platform to deliver and manage support programs to hundreds of participants simultaneously as in TEXT ME and TEXTMEDS trials. This research provides broad principles that may assist other researchers in developing mHealth programs.

## Introduction

The use of mobile phone technologies in health care has evolved into a new field of medicine known as mobile health (mHealth) [1]. Subscription to mobile phones is ever increasing with an estimated 7.1 billion mobile subscriptions and mobile network population coverage close to 95% [2]. Technology uptake is increasing among people across all socioeconomic classes [3,4], age groups [4], and continents [5]. Texting is a common mode of efficient, cheap, and personalized means of communication [6]. There is growing evidence on the role of text message-based programs for supporting health behavior changes [7-9] and improving adherence to treatment recommendations in the management of chronic diseases [10].

Despite emerging literature on the use of text message-based interventions for health care, there are a few explicit descriptions on the development of text message program content, structure, and message management software. A health researcher naïve to software-hardware complexities may have to rely entirely on an external professional agency; this carries the risk of inability to deliver the product to specifications, within budget or provide the product for long-term use. There are additional conceivable considerations such as legal obligations and privacy and security concerns over telecommunications-based programs.

**Figure 1:**
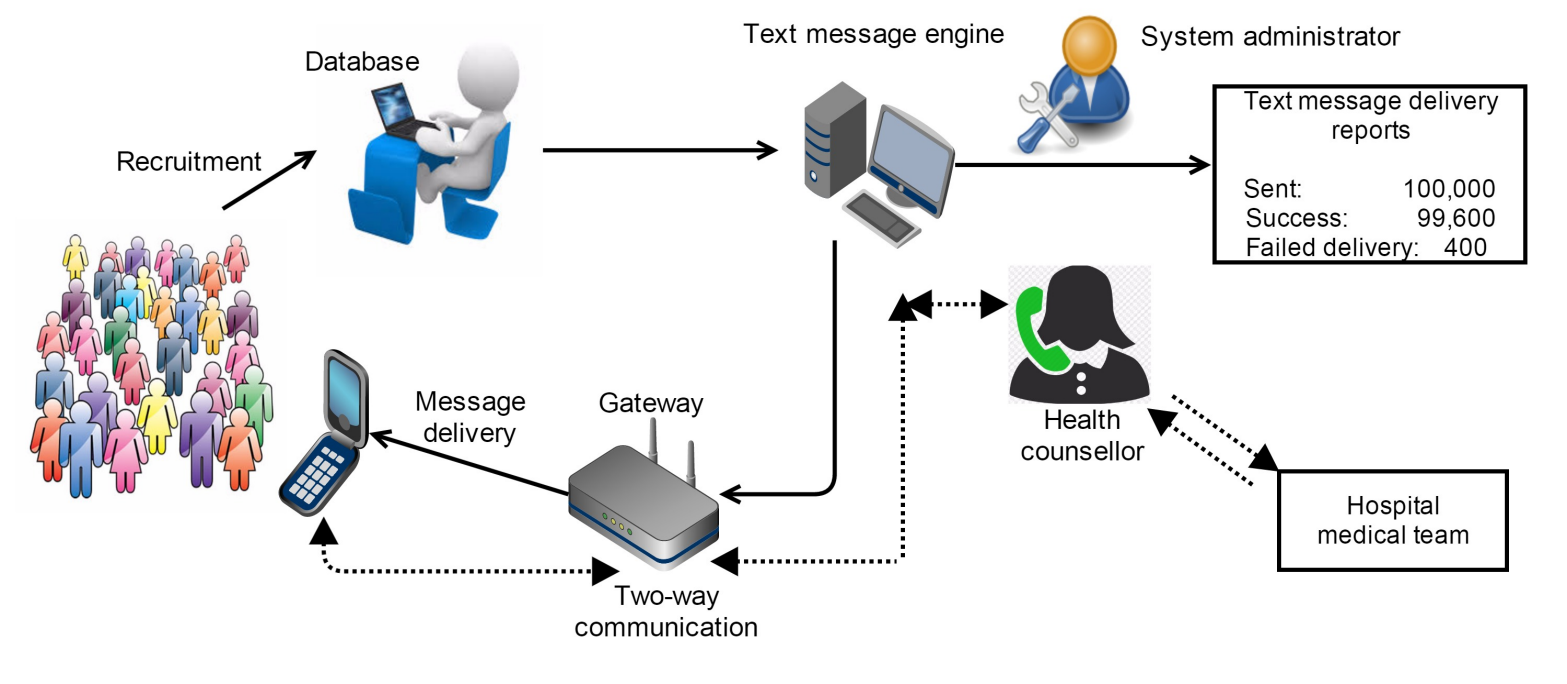
Staff, hardware infrastructure, and interactions for the TEXT messages to improve MEDication adherence & Secondary prevention (TEXTMEDS) study.

We have developed message management systems to support 2 large randomized controlled trials— Tobacco, EXercise and dieT Messages (TEXT ME; ACTRN 12611000161921) [11,12] and TEXT messages to improve MEDication adherence & Secondary prevention (TEXTMEDS; ACTRN 12613000793718) [13]. Both these trials were designed to evaluate cardiovascular disease secondary prevention support program delivered via mobile phone text messages to patients with coronary heart disease (CHD) (Table 1 and Figure 1). The aim of this paper was to outline the major practical elements that merit consideration when developing text message-based interventions. We do this by leveraging our experiences in developing the computerized message management system adopted in the TEXT ME and TEXTMEDS studies.

**Table 1:** Overview of TEXT ME and TEXTMEDS trials and intervention programs.

Study characteristics	TEXT ME	TEXTMEDS
Study design	Single blind^a^	Single blind^a^
	Randomized	Randomized
	Single center	Multicenter (Multitime zones)
	6 Month	12 Month
Primary focus	Behavioral change	Medication adherence
Sample size	700	1400
Message content	Lifestyle and general cardiovascular health advice	Medication adherence, lifestyle, and general cardiovascular health advice
Message delivery, time of the day	Randomly send, 10am-4pm	Randomly send, 10am-4pm
Message frequency	1 message per day; 4 random weekdays.	1 message per day; 2-4 random weekdays. Tailored frequency with fewer messages in the mid-trial compared with start and conclusion.
Program structure	Random message order. Each message is complete without reliance on previous content.	Structured delivery of information appropriate to the duration of the participant’s inclusion in the study.
Two-way communication	Not encouraged. Replies were monitored for regulatory compliance	Encouraged
Additional support	Not provided	Monthly reminders to participant about availability of health counselor for additional information

^a^Due to the nature of intervention, participants could not be blinded.

## Methods

We identified the following key stages in the developmental process for the message management system: (1) development of the program content (text message bank), (2) development of the text message engine software and integration with core database (participant data), and (3) text message send and gateway considerations. These are detailed in the following sections. In addition, we have described additional features that merit deliberation; text message identification codes, two-way communication considerations, text message reply monitoring, security-privacy, and legal requirements.

## Results

### Development of the Program Content (Text Message Bank)

There are a variety of approaches for developing program content; however, a systematic approach with the engagement of end-users is important. The process of development typically involves 3 phases: involving input from a range of experts and consumers, evaluation and refinement, and pilot testing. We have previously described the process of development of message content for the TEXT ME study [14]. During the first phase, a prototype bank was prepared by a multidisciplinary team incorporating various aspects, such as behavior change goals, scientific evidence and facts, and information from national health guidelines. The 160 character limit (including spaces) for a text message required careful wording and clarity of expression to avoid misunderstandings. In the second phase, the prototype bank was examined by practicing clinicians and potential consumers who reviewed each message using a survey that included questions with Likert-type responses about the readability, language appropriateness, and perceived utility of each message. The content was modified based on the survey feedback. The third phase involved pilot testing to ensure the functionality of the software, delivery of the text messages to recipients on different mobile networks, and seeking feedback on real-time experiences with respect to message frequency and timing. Following the pilot testing, further minor modifications were incorporated into the program prior to large-scale implementation of the main study. The final program structure and message content were based on the review of literature, feedback from potential participants, and pilot testing. When developing the text message program, it was essential to have a prespecified framework for how the content would be delivered and what participant information would be utilized to customize the messages. We considered a range of aspects to provide a support program, and enable future upscale of the program with minimal staff support (Table 2).

**Table 2:** Text message characteristics that merit consideration during the design phase.

Text message characteristic	Features	Features adapted in TEXT ME and TEXTMEDS studies
Message customization	The message content can be generic or individualized	Both studies sent messages with content that was partly generic and partly customized to participant’s needs eg, smoking status, diet (vegetarian or nonvegetarian) and types of medications.
	Ability to update custom settings. (particularly relevant for a long-duration study).	TEXT ME: Customization was only at baseline. TEXTMEDS: Allowed flexibility to changes in participant’s status such as behaviors or medication eg, if a participant successfully quit smoking and requested to stop smoking-related messages, this could be honored anytime during the 12-month study.
	Simple versus complex customization	TEXT ME: Relatively simple algorithms using minimal baseline data. TEXTMEDS: More complex customization using baseline data, and the ability to modify during the course of the program.
Personalization	This may enhance participant’s engagement with the program	Both studies implemented this function.
Delivery timing	Needs to consider intrusiveness of messages delivered during working hours, out off hours, weekends, and public holidays	Both studies sent messages on working days during working hours. Occasional season’s greetings message on holidays.
	Random times may prevent habituation.	Both studies implemented random delivery times on random weekdays.
	Specific times—delivery timed with a specific behavior eg, medication intake	We did not implement this aspect, as network latency times cannot be measured with confidence.
Frequency	Number of messages per day or per week	Both studies sent 1 message per day, average 4 messages per week.
	Fixed versus variable frequency	TEXT ME: A consistent schedule of 4 messages per week on random weekdays. TEXTMEDS: The frequency of messages varied from 2-4 per week on random weekdays.
Order of message content	Structured delivery may increase participant interest in program eg, patients hospitalized for myocardial infarction—early messages can focus on recovery and tips like use of prn nitrates; later messages can focus on healthy lifestyle, medium, and long-term goals	TEXT ME: Messages were written to stand on their own and not rely on previous messages. Hence, could be delivered in a random order. TEXTMEDS: Messages were delivered in order, enabling the ability to deliver a structured story. Messages delivered may reference a previous message. Participants received messages according to protocol-driven timing ie, on a given day each participant received a unique message, which may be different from other participants, but was appropriate to their duration on the study.
Unique messaging and repetition	Nonrepetitive messages, repetition of key messages or repetition of key message after rewording	TEXT ME had nonrepetitive messages TEXTMEDS had some repetitive key messages.
Two-way interaction	May increase patient engagement	TEXT ME was a one-way study. TEXTMEDS was two-way study. Participant replies were methodically logged and actioned by the health counselor.
Character set	Non-Latin (Unicode) characters are better avoided; alternatively, they must be tested for correct transmission by network operator and decoding by the recipient’s mobile phone	Both the studies had the capacity to support Unicode but did not implement this.
Readability	Avoidance of medical jargon and abbreviations	Both studies considered 5th-8th–grade reading level [15]. Text messages were pilot tested among prospective patients who were specifically asked about readability.
Message length	Preferred length ≤160 characters (including spaces)	In both studies the messages had a character count that ranged from 120-160. Longer messages are often delivered fractionated; consequentially may result in increased message send cost and unintelligible formatting by recipient’s handset.
Sender signature	Helps source recognition and distinguish the messages from spam. May be mandatory, refer to country-specific legislation	In both studies, participants were encouraged to save the study mobile number in the “contacts directory” of their mobile handsets. A full signature was included in the first message. Subsequent messages used abbreviated signature due to 160 character limit per text messages.
Unsubscription	Clear instructions for unsubscription; keyword - “STOP” or “OPT OUT” or “Unsubscribe”	Both studies included unsubscribe information with the first message. It was not possible to send unsubscribe information to participants with each message due length exceeding 160 characters. Legal advice recommended a way to consent participants so that this was not required.

### Considerations in Design of the Text Message Engine Software

A prerequisite to the development of any custom software is writing a “specifications” document to guide the programmers of the software. For example, in the TEXTMEDS study the key requirements identified included:

1. Automated import of data from a secure database into the text message engine software to minimize human errors from repetitive data entry.

2. Data validation of key variables, for example, mobile phone number and participant characteristics such as smoking status, diet, and medication class that guide customization.

3. Flexibility to accommodate changes in participant behavior during the course of the study.

4. The ability to receive text message replies from the participants as a summative digest in the form of a daily email to the health counselor.

5. Maintain chronological log for each participant transactions.

6. Include manual flexibility to send additional broadcast messages to all participants

7. Automated generation of daily encrypted backup files.

A precursor step for the delivery of a personalized and customized program was the necessity to assimilate participants “key characteristics” into the text message management software. This information can be entered manually into the software engine; however, this process remains vulnerable to human errors. To minimize such errors, simple measures can be implemented, for example, disabling copy-paste function and forced double-entry for the mobile phone number and other key variables that determine customization. Alternatively, the software can be configured to automatically import key data variables from a core database. With both the methods, a process of data validation is necessary and should be incorporated in the software.

### Text Message Send and Gateway

In order to send messages to the participants, the text message management software generally will have to integrate with a “Gateway.” In computer networking, a gateway is a nodal point that allows access to other networks; in this case, allowing Internet connected applications to access the participant’s telecom company. In both TEXT ME and TEXTMEDS, we contracted external companies to provide this service. When choosing a gateway for our studies, we considered a local reputable company that offered reasonable pricing, had the capacity to provide a log of delivery and failure reports, could capture participants’ replies, and forward them to our team. An important consideration is time taken by the Gateway to deliver a message, that is, latency. This can range from seconds to hours. Latency is primarily dependent on the type of network used and its ability to handle traffic during periods of heightened activity [16]. It is important to clarify this before engaging a prospective company and is especially relevant for a study that intends to deliver time-sensitive text messages, for example, timed with participant’s medications.

### Text Message Identification Codes

Telecom regulations require that the text messages should be sent with a unique code allocated to the sender. This confirms legitimacy and helps source identification. Some jurisdictions allow short codes (typically a 5-6 digit alpha-numeric), but most jurisdictions allow long codes or standard numeric phone numbers. The choice of the code (Table 3) is primarily dictated by the type of code available in the country, local regulations governing these codes, cost involved, and need for two-way communication. Dedicated short codes take a while for set-up, have substantial set-up fee, and often attract ongoing monthly fees. Premium services as mobile ringtone downloads, television program voting, and charity donations often use short codes. Reply messages sent to short codes by the customer are charged a higher fee and may even automatically subscribe a monthly fee to the recipient’s mobile phone account. As a result, many telecom companies have a default policy to block messages originating from the short codes. This can be a major hurdle for program implementation. By comparison, long code set-up is quicker, attracts conventional text message rates, and has the ability to reach all the carriers.

**Table 3:** Text message sending codes.

Code	Advantages	Disadvantages
Short code	Burst sending (30-40 text messages per second)	Easy to be mistaken as spam by the participant
	Easy to remember	Participants’ network provider may have a policy to block messages originating from a short code
		Limited to national borders
Long code	More likely to be acceptable and identifiable as genuine text message	Relatively slower message rate (1 message per second) depending on jurisdiction
	Long-term rental or lease can be cost effective	
	International reception capability	

### Two-Way Communication Considerations

Two-way communication may be incorporated into any text message-based study. These bilateral transactions may enhance participant engagement and can be a confirmation that a participant has read the text message. Bidirectional engagement can bolster trust in the patient-provider relationship [17]. Two-way communication, however, requires additional resources: monitoring of the “send-receive” loop, tracking of the conversation (to see which message is linked with which response), personnel to oversee and respond to messages, and the economic cost of replies via text message which must be either borne by the participant or charged back to the study.

Even if a study is designed to be one-way (eg, TEXT ME), it is important to monitor replies for regulatory compliance, for example, for unsubscribe requests. TEXTMEDS, by contrast, allowed and encouraged two-way communication. The TEXTMEDS software had the additional ability to file a chronologic log of all messages sent, the reply from the participant, and a corresponding response (if this was necessary) to the reply. This coherent conversation record is essential to enable tracking of the conversation with participants. Successful implementation of two-way communication requires a dedicated health counselor to monitor all the messages. The TEXMTEDS study counselors were trained to respond in accordance with a standardized manual. The health counselor role is probably best served by an allied health professional (eg, nurse or dietitian) and may need additional support from clinicians for specialist information. We did not implement “artificial intelligence” that is, the ability to automatically update settings and respond to text message responses from participants. This was because we were unsure about the frequency of participant replies and we expected a wide variation in responses; therefore, we considered but did develop this feature. The potential though remains intriguing as algorithmic analysis and computing power is rapidly evolving. This feature could be considered as a supplement to health counselor role in the next generation text message trials.

### Text Message “Send-Reply” Loop Monitoring

The breakdown in electronic communication loops is unpredictable and unfortunately not uncommon. Hence, all programs using two-way communication require some method to check the “send-receive” loop. This can be done manually or by using a virtual mobile. We utilized a second text message provider company that maintained a virtual mobile phone on our behalf. Each time a daily send occurred, we also sent a message (which we named the “heartbeat text message”) to the virtual mobile phone. It then sends an automated reply back to the primary text message provider, who is expected to send an email to our server registering the reply. If that reply is not received, the text message engine generates an error message to the study team. The “heartbeat text message” results in an additional cost but provides assurance that the loop is operational. Such monitoring is essential for large-scale program implementation. Part of our monitoring processes also involved checking if the messages were delivered uniformly across all major mobile phone service providers.

### Text Message Delivery Monitoring

It is important to configure text message management software to record delivery reports (success and failure) and generate an error advisory to the research staff. This is essential for monitoring the program, intervention fidelity analysis as well as early recognition of participants changing their mobile phone number. With some phone contracts, a phone number may be reallocated and messages may, therefore, be delivered to a different recipient. This may compromise privacy and confidentiality. Hence, it is essential to provide explicit instructions (eg, on a participant information sheet) at program initiation to promptly report any changes to their preferred mobile phone number and have a process in place that research staff recognizes this and execute immediate unsubscription till further contact with the participant is reestablished.

### Security Considerations

Data security and privacy should be of paramount importance in developing any technology-based programs. Security concerns arise at multiple levels in a text message program. Any application or business, no matter its purpose, is susceptible to hacking [18]. The electronic database, the text message engine software, communication portals between database-engine-gateway, and the text message itself are vulnerable to security and privacy compromise. The key consideration during the designing of any software is the necessity to protect information about the patient’s health, contact details, and all communication. The measures adopted in TEXT ME and TEXTMEDS studies are summarized in Table 4. A system backup was generated at regular intervals. With TEXT ME, the local computer maintained file backup of each state change in the data files. The hard drive was backed up to a local external drive after every operation (participant entry, parse, message-send). A weekly compact disk backup was created and stored offsite. With TEXTMEDS, the files were zipped into an archive and then encrypted using SHA256 hash function [19]. The encrypted study files were sent via email to the system administrator each day. The restore function was built into TEXTMEDS.

**Table 4:** Areas at risk of security compromise in a text message study and measures adapted in TEXT ME and TEXTMEDS study.

Components	Measures adapted in TEXT ME and TEXTMEDS study
Text message engine hardware	Access to room by authorized personnel only
	Unit physically locked to bench
Text message engine software	Dedicated computer for sole purpose of running text message engine
	Passwords at screen login and engine software login
	Front facing passwords are hashed and salted
	Computer is configured to prevent reboot from CD^a^ or USB^b^ drive
Database	Database selected with security levels required for holding potentially identifying patient data.
Communication between portals	Secure HTTP (hypertext transfer protocol) or secure sockets layer (SSL) emails
	Onsite and offsite data backup (encrypted)
Text message	Avoiding identifying information in the body of the text message
	Participants instructed to consider password protection of their mobile phone and disable message preview function

^a^CD: Compact disc.

^b^USB: Universal serial bus.

### Consideration of Legal Requirements

Text message communications are subject to 2 important laws—privacy policy and spam acts. It is important to note that approval by an ethics committee does not grant legislative exemption. The messages sent during the study (directly or indirectly) have a potential to disclose individual’s health information. Each country may have different legislation on privacy policy and data protection—Health Insurance Portability and Accountability ACT (HIPAA, USA) [20], Personal Information Protection and Electronic Documentation Act (PIPEDA, Canada), and Privacy Act 1988 (Australia) or Data Protection Directive (European Union). There are 2 possible approaches to mitigate this. First, restructure text messages to remove all personal health information; alternatively, retain limited personal information, but conduct a risk analysis to ensure proper security measures are executed [21].

Sending text messages in bulk is recognized as spam in most countries. Anti-spam legislation is governed in the United States by the CAN SPAM Act and Telephone Consumer Protection Act, and in Australia by the Spam Act [22,23]. Many countries have similar legislative requirements and these must be taken into consideration. Noncompliance penalties can be substantial. Texting in certain countries from certain institutions (eg, hospitals, schools, banks) may sometimes be categorized as transactional rather than commercial and awarded a special exemption status [24]. However, appropriate legal consultation where available should be solicited. Three key elements were identified to avoid legal consequences in our jurisdiction and are universally applicable. First, opt-in consent, that is, participants should provide written and explicit consent to receive messages corresponding to the trial duration. Second, each text message communication had to clearly identify the organization that authorized message send, that is, a unique signature for example, TEXT ME study used “TEXTME” and TEXTMEDS study used “TXTMED-abbreviated hospital name” as a sender signature. Third, opt-out capacity, that is, there had to be a clear method for unsubscription and this must be honored as soon as possible within legally acceptable time frame. It is important to note that as a standard text message length is of 160 characters length, including unsubscription information as well as health information within 160 characters was generally not possible. It was intended that this be highlighted to the participant at program initiation and included as a clause on the consent form as well as participant information sheet; thus, allowing us to avoid the necessity for explicit unsubscribe information with each text message.

## Discussion

### Principal Findings

This paper describes the key aspects that merit considerations in the design of an mHealth project. We have leveraged our experiences acquired during the development of the TEXT ME and TEXTMEDS text message management systems to provide a framework for other researchers planning similar projects. Automated message management systems can enable scalability, but should be developed and utilized in trial settings to obtain important information on their usability, reliability, and ability to deliver the intervention as intended.

To initiate the build of an mHealth intervention, there are 2 broad choices: (1) vendor-based solution or (2) In-house solution [25]. A vendor-hosted solution is usually a company (generally for profit) that offers Web-based solutions to their clients where the vendor essentially performs all functions other than writing content (eg, in the case of texting intervention—the messages) and pushing the “send” button. The advantages of this approach may include the relevant experience of the vendor, lack of requirements of up-front investment in software development, and efficient intervention delivery. Disadvantages may include high overall cost primarily determined by the extent of customization. It is important to determine the vendors track-record, service quality, privacy policy and policies on data sharing, and degree of security implemented by the vendor to protect participant’s personal information [26]. The alternative approach is an in-house solution. This allows maximum customization possibilities and desired functionality. The disadvantages of this approach include the need for technical expertise, large up-front cost in software development, lag time (software development to program initiation), and ongoing maintenance costs. While assessing the cost and benefits, researchers should also factor in—the duration of the study, size of study population, volume of text message exchange, and if there are plans to run parallel projects using the same software. The text message engine running the TEXT ME and TEXTMEDS studies were in-house designed, primarily because no appropriate vendor system existed (to our knowledge) that offered the level of customization we required. The team running these projects comprised of highly motivated researchers and academics with clinical and technical knowledge, which facilitated smooth delivery while keeping the overheads low. We also desired to deliver multiple similar trials varying by time zone, locations, and message customization [27].

Although this paper details the considerations and methodology relevant to developing a text message-based health intervention, there is a significant overlap with other mHealth interventions such as mobile device apps. The advantage of text message delivery include universal compatibility with all mobile handsets, requires minimal technological skills from participants and is a “push” technology (ie, it is delivered to the participant until they opt out). App-based interventions could be used to deliver a similar program with the potential advantages of lower delivery cost and the ability to deliver more graphical content. One potential advantage of app-based messaging is the ability to transmit highly secure information with end-to-end encryption. An app can also be designed to provide instantaneous automated responses to participant inputs. They also carry higher functionality, support interactivity, and bilateral engagement. The major disadvantages of app-based messaging approach are increased development cost, complexity, and compatibility issues that is, need for mobile phones or equivalent devices and Internet connectivity. This may be a limiting factor when the target audience comprises elderly or socioeconomically disadvantaged population. In addition, app-based interventions are more dependent on the user continuing to choose to use the app, whereas text message would continue to be delivered to the participant until they make an active choice to opt out of the program.

### Conclusions

Highly customized multifaceted interventions can be delivered to large patient populations using an automated text message engine. There is a need for further development of customized and structured text message programs that combines patient needs with the potential for large-scale delivery. Researchers running information technology projects may have constitutional obligations and must ensure reasonable steps for secure electronic communication.

## Abbreviations

CHD: coronary heart disease
HIPAA: Health Insurance Portability and Accountability ACT
HTTP: hypertext transfer protocol
mhealth: mobile health
PIPEDA: Personal Information Protection and Electronic Documentation Act
SSL: secure sockets layer
TEXT ME: Tobacco, EXercise and dieT Messages
TEXTMEDS: TEXT messages to improve MEDication adherence & Secondary prevention
